# Comparison of Patients With Familial Chylomicronemia Syndrome and Multifactorial Chylomicronemia Syndrome

**DOI:** 10.1210/clinem/dgae613

**Published:** 2024-09-06

**Authors:** Catherine M Spagnuolo, Jian Wang, Adam D McIntyre, Brooke A Kennedy, Robert A Hegele

**Affiliations:** Department of Medicine, Schulich School of Medicine and Dentistry, Western University, London, Ontario, Canada, N6A 5B7; Robarts Research Institute, Schulich School of Medicine and Dentistry, Western University, London, Ontario, Canada, N6A 5B7; Robarts Research Institute, Schulich School of Medicine and Dentistry, Western University, London, Ontario, Canada, N6A 5B7; Robarts Research Institute, Schulich School of Medicine and Dentistry, Western University, London, Ontario, Canada, N6A 5B7; Department of Medicine, Schulich School of Medicine and Dentistry, Western University, London, Ontario, Canada, N6A 5B7; Robarts Research Institute, Schulich School of Medicine and Dentistry, Western University, London, Ontario, Canada, N6A 5B7

**Keywords:** acute pancreatitis, familial chylomicronemia syndrome, hypertriglyceridemia, multifactorial chylomicronemia syndrome, pathogenic variant, type 1 hyperlipoproteinemia

## Abstract

**Context:**

Patients with rare familial chylomicronemia syndrome (FCS) and relatively common multifactorial chylomicronemia syndrome (MCS) both express severe hypertriglyceridemia, defined as plasma triglyceride concentration ≥10 mmol/L (≥885 mg/dL). Clinically there can be confusion between the 2 conditions.

**Objective:**

To compare clinical and biochemical phenotypes in patients with genotypically characterized FCS and MCS.

**Methods:**

We performed targeted sequencing of DNA from 193 patients with severe hypertriglyceridemia, classified them as having either FCS or MCS, and compared clinical and biochemical characteristics.

**Results:**

Patients with FCS were significantly younger than patients with MCS (31.4 ± 16.7 vs 51.0 ± 11.3 years; *P* = .003), with earlier age at symptom onset (15.0 ± 15.8 vs 37.8 ± 8.8 years; *P* = .00066), lower body mass index (23.3 ± 3.1 vs 30.7 ± 5.0 kg/m^2^; *P* = .000016), and higher prevalence of pancreatitis events (81.8% vs 35.2%; *P* = .003). Furthermore, patients with FCS had a higher ratio of triglyceride to total cholesterol (ie, 4.18 ± 0.92 vs 1.08 ± 0.51; *P* < .0001) and lower plasma apolipoprotein B (ie, 0.56 ± 0.15 vs 1.02 ± 0.43 g/L; *P* < .0001) than patients with MCS. Patients with MCS with heterozygous pathogenic variants had a relatively more severe clinical presentation than other MCS genetic subgroups.

**Conclusion:**

Patients with FCS have notable phenotypic differences from patients with MCS, although there is overlap. While genetic analysis of patients with persistent severe hypertriglyceridemia can definitively diagnose FCS, 8.8% of patients with MCS with sustained refractory hypertriglyceridemia behave functionally as if they have FCS, which should influence their eligibility for novel therapies for severe hypertriglyceridemia.

Familial chylomicronemia syndrome (FCS) and multifactorial chylomicronemia syndrome (MCS) are both characterized by severe hypertriglyceridemia (HTG), defined as a fasting plasma triglyceride (TG) concentration ≥10 mmol/L (≥885 mg/dL) (1-4). Severe HTG is seen in ∼1 in 400 North American adults (4), with >95% of cases being due to MCS (1, 3). Patients with severe HTG are at increased risk of severe recurrent and potentially fatal acute pancreatitis (1, 2). Novel pharmacologic therapies to reduce both TG levels and risk of acute pancreatitis in patients with severe HTG include inhibitors of apolipoprotein (apo) C-III (5-9). Access to such medications, once they are approved, may depend on specific genotypic and phenotypic characteristics of patients with severe HTG.

FCS is an extremely rare monogenic autosomal recessive condition that follows a biallelic inheritance pattern in which an individual inherits 2 pathogenic loss of function alleles, leading to complete absence of lipolytic activity (1, 10). The main plasma lipoprotein disturbance is excessive chylomicrons, with normal or even low levels of other lipoproteins. Patients present with severe HTG beginning in childhood. The estimated prevalence of FCS in the general population is 1 in 100 000 to 1 000 000 (1). To date, 5 canonical lipolysis genes are implicated in FCS, namely *LPL*, *GPIHBP1*, *APOA5*, *APOC2*, and *LMF1* (1).

In contrast, MCS is much more common, presenting in adulthood and often involving genetic predisposition, plus secondary factors such as poor diet, alcohol consumption, obesity, and type 2 diabetes (1-3, 11). The broader plasma lipoprotein disturbances in MCS include both excessive levels of chylomicrons, very–low-density lipoproteins and TG-rich remnants, with variable levels of other lipoproteins. The genetic component of MCS is complex, including contributions from single copies of pathogenic variants in 15% to 20% of cases, and/or polygenic risk from accumulated multiple common genetic variants (ie, single nucleotide polymorphisms [SNPs]) in 35-50% of cases (1, 11).

FCS and MCS are both severe dyslipidemias with high lifetime risk of acute pancreatitis, but the overall clinical severity of FCS is considered to be worse than MCS (1-3, 10-12). However, this has not yet been formally analyzed using genetic stratification that classifies individuals with FCS by biallelic pathogenic variants and MCS by either single copy heterozygous pathogenic variants, a high polygenic score, both, or neither. Here we used a hybrid targeted next-generation DNA sequencing panel to genetically define patients with FCS or MCS, and then compared clinical and biochemical features (13, 14).

## Materials and Methods

### Subjects

Patients were recruited from the Lipid Genetics Clinic at London Health Sciences Centre, University Hospital (London, ON, Canada) with the following inclusion criteria: patients aged ≥18 years and severe HTG defined as TG concentration ≥10 mmol/L (≥885 mg/dL) (11, 14). A clinical FCS score originally developed by Moulin et al (15) was also calculated for each participant. The Moulin score is a well-accepted clinical tool that reports the likelihood that a patient has FCS by assigning weights to clinical variables such as plasma TG >10 mmol/L (>885 mg/dL) measured on multiple occasions, refractory to standard TG-lowering therapies, a young age at onset, lack of secondary factors, and a history of episodes of acute pancreatitis (15). Patients with familial combined hyperlipidemia (former type 2B hyperlipoproteinemia), dysbetalipoproteinemia (former type 3 hyperlipoproteinemia), or with genetically confirmed partial lipodystrophy were excluded from this study. We also evaluated a subgroup of patients with MCS who were refractory to treatment, defined as persistent TG >10 mmol/L on at least 3 measurements, or, in other words, no recorded TG reading <10 mmol/L. Written informed consent was obtained from all patients. Informed consent and protocol were approved by the Western University Ethics Review Board (protocol #0379E).

### Biochemical Analyses

Fasting plasma lipid profiles and general biochemistry results were obtained as described (11).

### Genetic Analyses

Genomic DNA was extracted from peripheral blood and analyzed using a targeted resequencing panel for monogenic dyslipidemias as described (11, 13, 14). Gene sequencing was performed at the London Regional Genomic Centre at Robarts Research Institute (www.lrgc.ca) (13, 14). Briefly, next-generation sequencing of genomic DNA was carried out using the LipidSeq panel (14) to screen *LPL*, *GPIHBP1*, *APOA5*, *APOC2*, and *LMF1* genes and a set of 16 SNPs statistically associated with TG levels in the general population (16). Samples were enriched for the genomic area of interest and sequencing was performed using MiSeq personal sequencer (Illumina, San Diego, CA). FASTQ files derived from MiSeq output were processed using a customized workflow in CLC Bio Genomics Workbench (QIAGEN, Aarhus, Denmark) for sequence alignment against human reference genome (build hg19), variant calling and coverage statistics. Variant annotation was performed using ANNOVAR software (17) with customized scripts. Copy number variation analysis was performed using the VarSeq-CNV caller (Golden Helix, Bozeman, MT) (14). Pathogenicity classification of rare variants was performed under American College of Medical Genetics and Genomics guidelines (18). A weighted polygenic score comprised of 16 TG-associated SNPs was calculated as described (11)

### Statistical Analyses

The chi-square test was used to compare categorical/binary data between clinical and genotypic classes. Unpaired Student t-tests were used to compare continuous variables between clinical and genotypic classes. Multivariate logistic regression analysis was performed to determine clinical variables that significantly discriminated between patients with FCS and patients with MCS. All tests were 2-sided and *P* < .05 was deemed to be statistically significant. Statistical analyses were conducted using GraphPad Prism 10 (GraphPad Software, La Jolla, CA).

## Results

### Genotypes of Study Participants

Clinical and demographic data were collected from 193 patients from all follow-up appointments from 1997 to 2021. Patients had been followed between 2 and 23 years, with a minimum of 3 TG measurements (range 3-45 measurements), of which at least 1 was >10 mmol/L. For each participant, the historically highest and lowest TG measurements were evaluated and displayed graphically. Eleven patients had genetically proven FCS, defined as biallelic (ie, homozygous or compound heterozygous), rare loss of function pathogenic, or likely pathogenic (P/LP) variants in the 5 canonical lipolysis genes (19). Among the 11 patients with FCS, 3 were simple homozygotes, and 3 were compound heterozygotes for P/LP variants in *LPL*, 4 were simple homozygotes for P/LP variants in *APOC2*, 1 was a simple homozygote for a pathogenic variant in *APOA5*, and 1 was a compound heterozygote for pathogenic variants in *LMF1*. The P/LP variants in subjects with FCS are shown elsewhere (Table S1 (20)).

The remaining 182 patients were diagnosed with MCS. We subdivided patients with MCS into 5 genotypic subgroups based on heterozygosity for rare P/LP variant in 1 of the 5 canonical FCS genes, and/or presence of any rare variant identified in 1 of the canonical FCS genes or in the *CREB3L3* gene (currently not an officially recognized gene for FCS) (21), classified as a variant of uncertain significance or benign, and/or a high polygenic score for TG, defined as >90th percentile as described (11). Among 32 patients with MCS with single copy rare variants, 8, 1, and 1 had P/LP variants in *LPL*, *APOC2*, and *APOA5*, respectively; 10 had a single variant of uncertain significance in either *LPL*, *APOC2*, *APOA5*, or *LMF1*; 7 had 1 or 2 rare variants in *CREB3L3*, and 3 had a single copy rare benign variant in *LMF1.* Details of the rare variants in subjects with MCS are shown elsewhere (Table S1 (20)). In addition, 80 of 182 subjects with MCS had a polygenic score for TG >90th percentile, a remarkable excess (more than 4-fold) of polygenic risk. Nine patients with MCS had both a heterozygous rare variant plus a high polygenic score for TG, while 88 patients with MCS had neither a heterozygous rare variant nor a high polygenic score for TG.

The 5 MCS genotypic subgroups were as follows: (1) MCS-monogenic (n = 32), in which patients carried a single rare P/LP variant; (2) MCS-polygenic (n = 71), in which patients had a high polygenic score for TG defined as >90th percentile; (3) MCS-monogenic and polygenic (n = 9), in which patients had both a P/LP rare variant and a high polygenic score; (4) MCS with either genetic factor (n = 94); and (5) MCS with no identified genetic factor (n = 88). Rare variants identified in patients and the American College of Medical Genetics and Genomics classifications of the variants are listed elsewhere (Table S1 (20)).

### Comparison of Phenotypes of Patients With FCS and Patients With MCS

Table 1 summarizes demographic and clinical characteristics of study participants. Patients with FCS were significantly younger (*P* = .003) than patients with MCS, with earlier symptom onset (*P* = .00066) and lower body mass index (BMI, *P* = .000016), and were less likely to have European ancestry (*P* < .0001).

**Table 1.** Characteristics of patients with familial chylomicronemia syndrome in comparison to patients with multifactorial chylomicronemia syndrome

	FCS patients	MCS patients	*P* value
Number	11	182	
Age at enrolment (years, mean ± SD)	31.4 ± 16.7	51.0 ± 11.3	.003
Age of symptom onset (years, mean ± SD)	15.0 ± 15.8	37.8 ± 8.8	.00066
Sex, % of females	27.3	28.6	NS (>.99)
BMI (kg/m^2^, mean ± SD)	23.3 ± 3.1	30.7 ± 5.0	.000016
European ancestry (self-declared) (%)	45.5	94.0	<.0001
Peak TG (mmol/L, mean ± SD)	44.2 ± 12.1	26.7 ± 21.6	.0006
Lowest TG (mmol/L, mean ± SD)	9.78 ± 4.36	3.93 ± 3.62	.001
Lowest measured TG >10 mmol/L (%)	45.5	8.8	.002
Lowest measured TG <2 mmol/L (%)	0	33.0	.019
Peak TC (mmol/L, mean ± SD)	10.5 ± 2.9	9.98 ± 1.75	NS (.63)
Lowest TC (mmol/L, mean ± SD)	3.68 ± 1.37	4.52 ± 1.75	NS (.16)
TG/TC ratio (mmol/L, mean ± SD)	4.18 ± 0.92	1.08 ± 0.51	<.0001
TG/TC ratio >3.52 (%)	72.7	0	<.0001
Apo B (g/L, mean ± SD)	0.56 ± 0.15	1.02 ± 0.43	<.0001
Peak HDL-C (mmol/L, mean ± SD)	0.65 ± 0.30	1.20 ± 0.39	.014
Lowest HDL-C (mmol/L, mean ± SD)	0.35 ± 0.11	0.77 ± 0.86	<.0001
Ever on fibrate treatment (%)	90.9	85.3*^a^*	NS (>.99)
Ever on fibrate + another LLM*^a^* (%)	63.6	66.7*^a^*	NS (>.99)
Ever on nonfibrate LLM*^b^* (%)	0	11.9*^a^*	NS (.61)
Previous episode of acute pancreatitis (%)	81.8	35.2	.003
Recurrent pancreatitis (%)	72.7	17.6	.0002
Type 2 diabetes mellitus (%)	0	39.6	.008
Hypertension (%)	9.1	49.1*^a^*	.011
Smoking history (%)	18.2	31.5*^a^*	NS (.51)
Alcohol consumption (%)	27.3	63.0*^a^*	.026
Cardiovascular disease history (%)	0	9.1*^a^*	NS (.59)
Moulin FCS score (mean ± SD)	12.4 ± 1.9	2.6 ± 4.4	<.0001
Moulin FCS score ≥ 10 (%)	100	8.8	<.0001

Abbreviations: Apo, apolipoprotein; BMI, body mass index; FCS, familial chylomicronemia syndrome; HDL-C, high-density lipoprotein cholesterol; LLM, lipid-lowering medication; MCS, multifactorial chylomicronemia syndrome; NS, nonsignificant; TC, total cholesterol; TG, triglyceride.

^*a*^Case number less than 182 in MCS group: n = 177 for ever on fibrate treatment, ever on fibrate + another lipid-lowering medication, and ever on nonfibrate lipid-lowering medication; n = 173 for hypertension; n = 149 for smoking history; n = 135 for alcohol consumption; n = 66 for cardiovascular disease.

^*b*^Nonfibrate lipid-lowering medication includes statin, ezetimibe, niacin, and/or omega-3 fatty acids.

Among biochemical features, patients with FCS had higher peak and trough TG levels (*P* = .0006 and .001, respectively) than patients with MCS, with a higher proportion having lowest TG >10 mmol/L (45.5% vs 8.8%, *P* = .002). No patient with FCS had a recorded lowest measured plasma TG <2 mmol/L compared with 33.0% of patients with MCS (*P* = .019). In contrast, peak and trough levels of total cholesterol (TC) did not differ between patients with FCS and patients with MCS.

In addition, patients with FCS had a higher TG/TC ratio (ie, 4.18 ± 0.92 vs 1.08 ± 0.51; *P* < .0001) and lower plasma apo B level (ie, 0.56 ± 0.15 vs 1.02 ± 0.43 g/L; *P* < .0001) than patients with MCS. In patients with FCS, 72.7% had a TG/TC ratio >3.52 mmol/L vs 0% of patients with MCS (*P* < .0001). Furthermore, peak and trough levels of high-density lipoprotein cholesterol (HDL-C) were significantly lower in patients with FCS than in patients with MCS (*P* = .014 and <.0001, respectively).

There were no significant differences between patients with FCS and patients with MCS with respect to history of fibrate and/or other lipid-lowering therapy including statins, ezetimibe, niacin, and/or omega-3 fatty acids. Acute pancreatitis history was higher in patients with FCS than in patients with MCS (ie, 81.8% vs 35.2%; *P* = .003). Patients with FCS were also more likely to have experienced recurrent episodes of pancreatitis than patients with MCS (ie, 72.7% vs 17.6%, respectively; *P* = .0002).

With respect to secondary factors associated with HTG, patients with MCS were significantly more likely to (1) consume any alcohol than patients with FCS (ie, 63.0% vs 27.3%; *P* = .026); (2) have type 2 diabetes mellitus (ie, 39.6% vs 0%; *P* = .008); and (3) have hypertension (ie, 49.1% vs 9.1%; *P* = .011). There was no significant difference between patients with FCS and patients with MCS regarding history of smoking or cardiovascular disease.

Patients with FCS had a higher Moulin FCS score than patients with MCS (12.4 ± 1.9 vs 2.6 ± 4.4, *P* < .0001). Furthermore, 100% of patients with FCS had Moulin scores ≥10 compared with only 8.8% of patients with MCS (*P* < .0001).

### Multivariate Regression Analysis

Multivariate regression analysis showed that 4 clinical or biochemical features were significantly associated with FCS status: TG/TC ratio >3.52, a low BMI, younger age, and lowest recorded TG reading >10 mmol/L (Table 2). Other variables were not significantly associated.

**Table 2.** Multivariate regression analysis of clinical and biochemical variables in patients with familial chylomicronemia syndrome vs patients with multifactorial chylomicronemia

Variable	Beta-coefficient	SE	t-statistic	*P* value
Intercept	0.0703	0.166	0.423	NS (.673)
TG/TC >3.52	0.776	0.058	13.37	3.51×10^−28^
Body mass index	0.0066	0.0020	3.375	.0009
Age	0.0021	0.00080	2.661	.009
Lowest TG >10 mmol/L	0.0933	0.0312	2.994	.0032
Lowest TG <2 mmol/L	−0.0281	0.0210	−1.343	NS (.181)
Male sex	−0.024	0.0214	−1.1028	NS (.272)
European ancestry	−0.0513	0.0364	−1.409	NS (.160)
Apo B	0.0235	0.0216	1.089	NS (.278)
Diabetes	−0.0074	0.0204	−0.361	NS (.719)
Hypertension	0.0124	0.0195	0.633	NS (.528)
Smoking	−0.0094	0.0200	−0.480	NS (.632)
Alcohol use	−0.0096	0.021	−0.459	NS (.647)

Abbreviations: Apo, apolipoprotein; NS, nonsignificant; TC, total cholesterol; TG, triglyceride.

### Plasma TG Ranges by Genotypic Group

**Figure 1.**
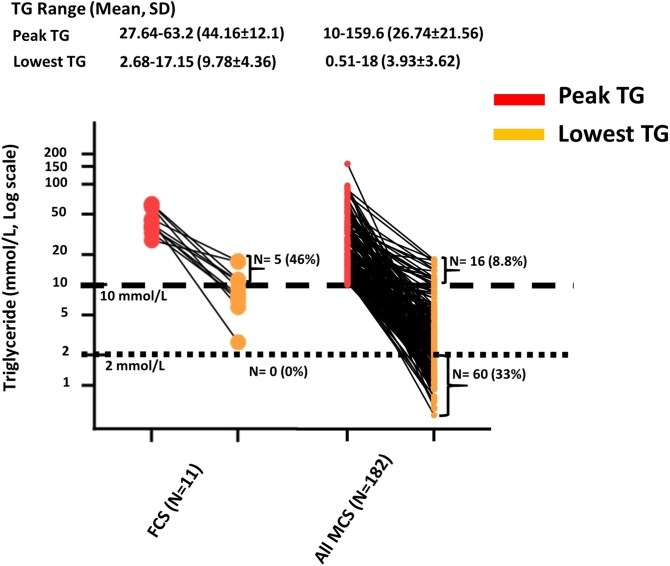
Triglyceride range in patients with familial chylomicronemia syndrome in comparison to patients with multifactorial chylomicronemia syndrome. Peak (maximum) and trough (minimum) triglyceride levels are shown. Abbreviations: FCS, familial chylomicronemia syndrome; MCS, multifactorial chylomicronemia syndrome; TG, triglyceride.

**Figure 2.**
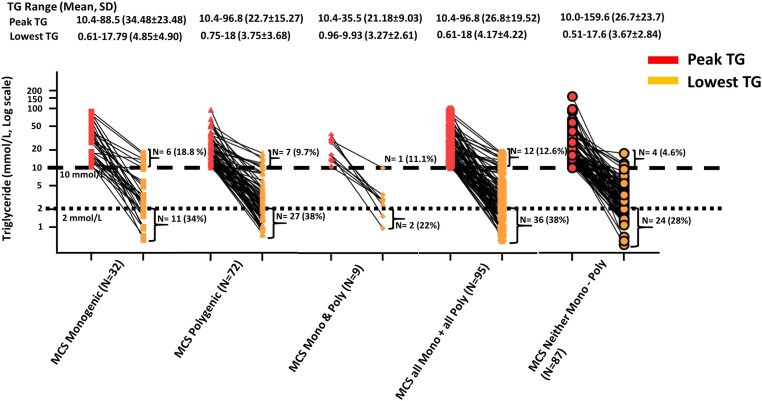
Triglyceride range in patients with multifactorial chylomicronemia syndrome by genotypic subgroup. Peak (maximum) and trough (minimum) triglyceride levels are shown. Abbreviations: MCS, multifactorial chylomicronemia syndrome; MCS-monogenic, patients with MCS heterozygous for rare pathogenic variant or likely pathogenic variant in 1 of the 5 canonical lipolysis genes and/or with any rare genetic variant in 1 of the canonical lipolysis genes or in the *CREB3L3* gene; MCS-mono & poly, patients with MCS with any rare genetic variant and a high polygenic score; MCS-polygenic, patients with MCS with a high polygenic score; MCS all mono + all poly, patients with MCS with any genetic cause; MCS neither mono-poly, patients with MCS with no genetic cause; TG, triglyceride.

The ranges of recorded peak and trough TG levels for individual patients are shown in Figs. 1 and 2. Statistical comparisons by clinical and genetic subgroups are shown elsewhere (Tables S2, S3, and S4 (20)). Figure 1 shows data for all patients with FCS and patients with MCS. Pairwise statistical comparisons of all patients with FCS vs all patients with MCS showed significantly higher peak and trough TG levels in patients with FCS (Table S2 (20)), as well as lower proportions with trough TG <10 mmol/L (Table S3 (20)) and trough TG <2 mmol/L (Table S4 (20)). Almost half of patients with FCS had no TG reading <10 mmol/L; the rest achieved levels between 2 and 9.9 mmol/L, and none ever achieved a TG level <2 mmol/L. In contrast, one-third of patients with MCS achieved essentially normal TG levels <2 mmol/L at some point in their care while only 8.8% failed to show a TG reading <10 mmol/L; both these differences between patients with FCS and patients with MCS were statistically significant (Tables S3 and S4 (20)).

Figure 2 shows the individual maximum and minimum TG levels for MCS genotypic subgroups (ie, MCS-monogenic; MCS-polygenic; MCS-monogenic + polygenic; MCS-monogenic or polygenic, and MCS-no genetic cause). Statistical comparisons of these values by clinical and genetic subgroups are shown elsewhere (Tables S2 to S4 (20)). Peak and trough TG levels were not significantly different among MCS genotypic subgroups, although all mean TG levels in genotypic subgroups were significantly lower than in patients with FCS (Table S2 (20)). Among the MCS subgroups, 18.8%, 9.7%, 11.1%, 12.6%, and 3.5% had trough TG levels >10 mmol/L, which did not differ statistically from each other or from the overall 8.8% proportion of patients with MCS, although most individual subgroup proportions were significantly lower than 45.5% for patients with FCS (Table S3 (20)).

Furthermore, among the MCS-monogenic, MCS-polygenic, MCS-monogenic + polygenic, MCS-monogenic and polygenic, and MCS-no genetic cause subgroups, 34.4%, 37.5%, 22.2%, 37.9% and 27.6%, respectively, had trough TG levels <2 mmol/L (Fig. 2). Again, these did not differ statistically from each other or from the 33.0% proportion overall of patients with MCS who attained TG levels <2 mmol/L, although each subgroup is statistically different from the 0% of patients with FCS who attained TG levels <2 mmol/L (Table S4 (20)).

### Patients With MCS With Refractory or Sustained Hypertriglyceridemia

We evaluated the subset of patients with MCS with TG never <10 mmol/L. We compared these individuals, who constituted 8.2% of the MCS cohort, with the remainder of the MCS cohort (Table 3). These individuals had significantly higher peak and trough TG levels, with no occurrences of TG <2 mmol/L, higher trough TC levels, lower peak HDL-C levels, and significantly higher Moulin scores, although only a minority (37.5%) had a Moulin score >10 compared with only 6.02% of others with MCS. Also, 37.5% of those with refractory or sustained HTG had a heterozygous pathogenic variant compared with 15.7% of others with MCS (*P* = .04). There was no significant difference in the proportion of those in this refractory subgroup who had a high polygenic score for TG (43.8% vs 39.2%, *P* = .79).

**Table 3.** Characteristics of patients with refractory or sustained chylomicronemia compared with others with multifactorial chylomicronemia syndrome

	Refractory MCS patients	Other MCS patients	*P* value
Number	16	166	
Age at enrolment (years, mean ± SD)	47.6 ± 12.0	51.6 ± 11.2	NS (.22)
Age of symptom onset (years, mean ± SD)	36.6 ± 6.6	38.0 ± 9.2	NS (.57)
Sex, % of females	31.3	28.3	NS (.78)
BMI (kg/m^2^, mean ± SD)	33.1 ± 4.3	30.5 ± 5.0	.029
European descent (%)	100	93.4	.602
Peak TG (mmol/L, mean ± SD)	39.0 ± 20.1	25.6 ± 21.4	.020
Lowest TG (mmol/L, mean ± SD)	13.6 ± 2.80	3.00 ± 1.96	6.9 × 10^−11^
Lowest measured TG >10 mmol/L (%)	100	0	<.00001
Lowest measured TG <2 mmol/L (%)	0	36.1	.0016
Peak TC (mmol/L, mean ± SD)	13.6 ± 7.94	9.63 ± 9.31	.074
Lowest TC (mmol/L, mean ± SD)	7.30 ± 2.51	4.26 ± 1.41	.0006
TG/TC ratio (mmol/L, mean ± SD)	1.29 ± 0.59	1.06 ± 0.5	NS (.15)
TG/TC ratio >3.52 (%)	0	0	NS (1.0)
Apo B (g/L, mean ± SD)	1.2 ± 1.1	1.0 ± 0.30	NS (.51)
Peak HDL-C (mmol/L, mean ± SD)	0.93 ± 0.39	1.22 ± 0.38	.011
Lowest HDL-C (mmol/L, mean ± SD)	0.61 ± 0.21	0.79 ± 0.89	NS (.069)
Ever on fibrate treatment (%)	62.5	84.9	.034
Ever on fibrate + another LLM*^a^* (%)	43.8	66.9	NS (.097)
Ever on nonfibrate LLM*^a^* (%)	18.8	10.8	NS (.40)
Previous episode of acute pancreatitis (%)	56.3	33.1	NS (.10)
Recurrent pancreatitis (%)	31.3	16.3	NS (.16)
Type 2 diabetes mellitus (%)	50.0	38.6	NS (.43)
Hypertension (%)	50.0	46.4	NS (.80)
Smoking history (%)	37.5	48.8	NS (.44)
Alcohol consumption (%)	18.8	49.4	.033
Cardiovascular disease history (%)	6.3	3.0	NS (.43)
Moulin FCS score (mean ± SD)	7.4 ± 3.1	2.1 ± 4.2	3.3 × 10^−6^
Moulin FCS score ≥ 10 (%)	37.5	6.02	.0008

Abbreviations: Apo, apolipoprotein; BMI, body mass index; FCS, familial chylomicronemia syndrome; HDL-C, high-density lipoprotein cholesterol; LLM, lipid-lowering medication; MCS, multifactorial chylomicronemia syndrome; NS, nonsignificant; TC, total cholesterol; TG, triglyceride.

## Discussion

We found that compared with patients with MCS, patients with FCS had (1) lower BMI; (2) younger age at assessment and earlier symptom onset; (3) higher likelihood of non-European ancestry; (4) higher TG levels at all stages, including greater proportions of those with TG that were never <10 mmol/L or <2 mmol/L; (5) higher baseline ratio of TG to TC, as well as lower apo B and HDL-C levels; (6) higher prevalence of episodes of acute pancreatitis; and (7) lower prevalence of secondary factors such as alcohol use and diabetes. Other variables, such as TC level, use of lipid-lowering medications, and history of smoking or cardiovascular disease, did not differ between FCS and MCS groups. Multivariate regression analysis identified the 4 variables that were most significantly associated with FCS compared with MCS status: (1) higher TG/TC ratio (cut point >3.52); (2) lower BMI; (3) younger age of symptom onset; and (4) trough TG level >10 mmol/L. Finally, there were no differences in TG-related variables across genetic subtypes of MCS. We also found that a subgroup of 8.8% of patients with MCS with persistent TG >10 mmol/L had higher BMI, higher TG, and were more likely to have a heterozygous P/LP variant than other patients with MCS. Overall, the findings are consistent with a more severe metabolic impairment associated with the genetically determined lipolytic deficiency from conception in patients with FCS compared with the relative deficiency of lipolysis in patients with MCS, which can become saturated over time with accretion of extrinsic secondary factors.

Our findings confirm and expand upon earlier observations in other cohorts, which generally reported that patients with FCS compared with patients with MCS had younger age at diagnosis, lower BMI, lower blood pressure, plasma glucose, and HDL-C, and a higher prevalence of abdominal pain and pancreatitis (22-25). Those studies found negligible differences between patients with FCS and patients with MCS with respect to baseline plasma TC and TG levels while plasma apo B was typically not determined. In contrast, our study found that a high TG/TC ratio and low plasma apo B were among the strongest discriminators between FCS and MCS. The characteristic high TG/TC ratio and low apo B levels in patients with FCS reflect isolated chylomicronemia combined with paucity of other lipoprotein and remnant species (1, 2). In contrast, the relatively low TG/TC ratio and high apo B in patients with MCS reflect a much broader range of lipoprotein disturbances, including greater quantities of cholesterol-enriched and apo B–containing lipoprotein species, such as very–low-density lipoproteins and their remnants. This is typical of Fredrickson hyperlipoproteinemia type 5, an archaic term previously used to describe patients with MCS. TG/TC ratio and/or apo B level could arguably be included in a revised clinical scoring algorithm to further enhance the ability to discriminate between FCS and MCS. Because apo B is not routinely available, the TG/TC ratio would seem to be the more practical clinical variable.

Some variables that we evaluated are already included in clinical scoring algorithms for FCS, such as the Moulin score (15), specifically (1) sustained fasting TG >10 mmol/L; (2) failure to attain TG <2 mmol/L; (3) a history of pancreatitis, and (4) younger age of onset. It is thus not surprising that we found significant differences between patients with FCS and patients with MCS both in the absolute Moulin score (ie, 12.4 ± 1.9 vs 2.6 ± 4.4; *P* < .0001), and in the proportion of patients with FCS vs patients with MCS with “very likely FCS” who scored ≥10 points (ie, 100% vs 8.8%; *P* < .0001). Our findings support the Moulin score as a predictor of genotypic FCS vs MCS.

Almost half of patients with FCS had no recorded TG <10 mmol/L, while the rest achieved TG levels between 2 and 9.9 mmol/L, although none achieved TG <2 mmol/L. In contrast, one-third of patients with MCS achieved TG <2 mmol/L at some point, while 8.8% never had TG <10 mmol/L. This latter refractory subgroup, while having genotypic MCS (by virtue of lack of biallelic P/LP variants and either heterozygosity, high polygenic score, both or neither) defines a clinically relevant “sustained” or “refractory” HTG subgroup of patients with chylomicronemia. Although they do not have genotypic FCS, this MCS subgroup are refractory to interventions and are at increased risk of acute pancreatitis. It remains possible that these patients actually have FCS because of a undetectable second pathogenic variant, or because they have either a novel causal gene, or novel pathogenic mechanism such as epigenetic imprinting or mitochondrial DNA inheritance. Nevertheless, according to current knowledge and technology, they are patients with MCS and their clinical need rather than genotype should be the primary determinant of access to effective novel therapies such as inhibitors of apo C-III.

It is of interest that a higher percentage of those with an identified genetic factor (either heterozygous P/LP variant, high polygenic score for TG, or both) have refractory HTG compared with those with no identified genetic factor (ie, 12.6% vs 3.5%; *P* = .035). Also, the highest percentage of refractory patients (18.5%) was in the MCS-monogenic subgroup with a heterozygous P/LP variant. However, all MCS genetic subgroups had some patients with refractory HTG, suggesting that genetic analysis is not definitive for this clinical state. Again, for clinical purposes the refractory nature of the severe HTG phenotype—and not the genotype—should be primary determinant of access to therapy.

Our data also suggest that the differences between genotypic subgroups of MCS are otherwise statistically and clinically nonsignificant (Fig. 2). While this could be related to small numbers of subjects in certain genetic subgroups, it reinforces current advice that genotyping and genetic stratification are not clinically helpful in most cases of MCS (26). For instance, substantial proportions of each MCS genetic subgroup attained TG levels <2 mmol/L, which did not differ statistically from each other or from the 33.0% proportion overall of patients with MCS who attained such levels. However, each MCS subgroup was difference significantly from the 0% of patients with FCS who attained TG levels <2 mmol/L. These findings also confirm that MCS is more responsive than FCS to current TG-lowering strategies including diet and lifestyle modification, fibrates, and/or omega-3 fatty acids.

Our study was limited overall by relatively small sample size, especially of the FCS cohort and genetic subgroups of MCS. Ideally, future multicenter studies will allow for recruitment of larger patient numbers to help identify additional differences in either individual or combinations of clinical and biochemical variables.

In summary, several clinical and biochemical features differ between patients with FCS and patients with MCS, including an elevated TG/TC ratio and low apo B in FCS compared with MCS cohorts. These variables could be used in non-DNA–based diagnostic algorithms. Genetic subtyping of patients with MCS according to presence of a heterozygous P/LP variant and/or a high polygenic score for TG did not reveal marked between-group differences in clinical or biochemical features. Finally, 8.8% of patients with MCS had refractory or sustained HTG. These patients, together with genotypically confirmed patients with FCS, might be potentially eligible for access to emerging advanced TG-lowering medications, such as apo C-III inhibitors.

## Data Availability

Some or all datasets generated during and/or analyzed during the current study are not publicly available but are available from the corresponding author on reasonable request.

## Abbreviations

apo: apolipoprotein
BMI: body mass index
FCS: familial chylomicronemia syndrome
HDL-C: high-density lipoprotein cholesterol
HTG: hypertriglyceridemia
MCS: multifactorial chylomicronemia syndrome
SNP: single nucleotide polymorphism
P/LP: pathogenic or likely pathogenic
TC: total cholesterol
TG: triglyceride
